# Insights into the Dynamics of the Human Zinc Transporter ZnT8 by MD Simulations

**DOI:** 10.1021/acs.jcim.0c01139

**Published:** 2021-01-29

**Authors:** Davide Sala, Andrea Giachetti, Antonio Rosato

**Affiliations:** † Magnetic Resonance Center (CERM), University of Florence, Via Luigi Sacconi 6, 50019 Sesto Fiorentino, Italy; ‡ Consorzio Interuniversitario di Risonanze Magnetiche di Metallo Proteine, Via Luigi Sacconi 6, 50019 Sesto Fiorentino, Italy; § Department of Chemistry, University of Florence, Via della Lastruccia 3, 50019 Sesto Fiorentino, Italy

## Abstract

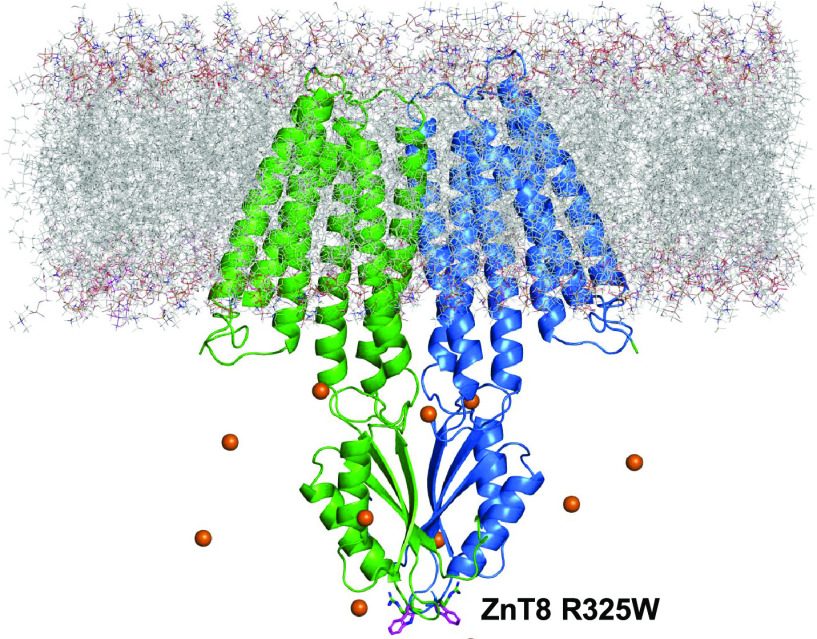

ZnT8 is a human zinc(II) transporter expressed at the membrane of secretory granules where it contributes to insulin storage importing zinc ions from the cytosol. In the human population, the two most common ZnT8 variants carry an arginine (R325) or a tryptophan (W325) in position 325. The former variant has the most efficient kinetics in zinc transport and has been correlated to a higher risk of developing insulin resistance. On the contrary, the W325 variant is less active and protects against type-2-diabetes. Here, we used molecular dynamics (MD) simulations to investigate the main differences between the R325 and W325 variants in the interaction with zinc(II) ions. Our simulations suggested that the position of the metal ion within the transport site was not the same for the two variants, underlying a different rearrangement of the transmembrane (TM) helices in the channel. The W325 variant featured a peculiar zinc environment not detected in the experimental structures. With respect to conformational dynamics, we observed that the R325 variant was significantly more flexible than W325, with the main role played by the transmembrane domain (TMD) and the C-terminal domain (CTD). This dynamics affected the packing of the TM helices and thus the channel accessibility from the cytosol. The dimer interface that keeps the two TM channels in contact became looser in both variants upon zinc binding to the transport site, suggesting that this may be an important step toward the switch from the inward- to the outward-facing state of the protein.

## Introduction

Zinc is a key element for all kingdoms of life and is ubiquitous within cells, where it can be found in the cytoplasm, nucleus, and organelles.^1^ Many important cellular functions require zinc, such as gene expression, DNA synthesis, immune response, hormone storage and release, neurotransmission, memory, and apoptosis.^2^ It has been estimated that approximately 10% of human proteins bind one or more zinc ions as a catalytic or structural cofactor.^3^ The correct zinc concentration in cells and organelles is maintained by a fine-tuned homeostasis regulation system.^4−6^ Specific protein families transport zinc ions inside or outside cells and cellular compartments. Proteins of the cation diffusion facilitator (CDF) family remove zinc from the cell or from organelles, whereas ZRT/IRT-like proteins (ZIPs) are responsible for zinc uptake in the cytoplasm from extracellular space and intracellular compartments.^7−9^ Members of both families feature tissue-specific expression and respond differentially to zinc overload and deficiency.^10,11^ Humans have 14 ZIP/Slc39 proteins and a specific CDF subfamily named ZnT/Slc30 of which 10 members are known so far.^12^ ZnT proteins can be separated into four groups based on their sequence similarity.^4^ Among them, ZnT2, ZnT3, ZnT4, and ZnT8 belong to subfamily 2, which transports zinc ions from the cytosol into intracellular vesicles. In particular, ZnT8 (SLC30A8) is localized to the membrane of insulin secretory granules of pancreatic β-cells, where it mediates zinc enrichment by importing ions from the cytosol.^13^ Inside secretory granules, zinc triggers insulin storage through the formation of insoluble hexamers in which insulin molecules are complexed with zinc and calcium ions in a 6:2:1 stoichiometry.^14,15^ Defects in insulin secretion have been linked to an altered risk of developing type-2 diabetes (T2D).^16^ ZnT8 might also be involved in glucagon release from pancreatic α-cells.^17^ A nonsynonymous single nucleotide polymorphism in the ZnT8 gene causes the replacement of arginine with tryptophan in position 325. The resulting variants have a different prevalence in the human population, with the most common R235 in a range of 60–95% and the less common W325 in a range of 5–40%.^18^ Which variants confer protection against T2D has been a matter of debate. Less frequent variants with modest effects had been initially associated with an increased risk of developing T2D.^19,20^ More recently, a number of loss-of-function mutations were correlated to protection against diabetes.^21^ In studies of zinc transport activity, using the radioactive isotope ^65^Zn in human embryonic kidney cells (HEK293), no detectable differences were found between the high-risk R325 and low-risk W325 variants as well as between two W325 isoforms differing for the presence of a 49 residues N-terminal extension.^22^ Instead, in proteoliposomes, the R325 variant exhibited higher zinc transport activity than W325 and has been correlated with an increased risk of T2D.^23^

Recently, the first cryo-electron microscopy (cryo-EM) structures of the ZnT8 protein have been solved.^24^ This, in combination with structural and functional studies on the prokaryotic CDF homologues, provides insights into the structure and function of the family.^25−27^ Studies on the YiiP transporter from *Escherichia coli* revealed a cation–proton antiporter exchange mechanism in which a zinc(II) ion is exported outside the cell, and a proton is imported in a 1:1 stoichiometry.^28^ The outward-facing (OF) state of the YiiP protein was characterized by X-ray crystallography revealing a Y-shaped homodimer structure, common to other CDF proteins, consisting of a transmembrane domain (TMD) connected to a C-terminalcytosolic domain (CTD).^29,30^ Each arm of the TMD consists of six transmembrane (TM) helices grouped in a four-helix bundle (TM1–TM2–TM4–TM5) and a helix pair (TM3–TM6). The TM3–TM6 helix pair connects the TMD to the cytosolic region and is splayed apart in the outward-facing state, giving the Y shape to the YiiP transporter. In addition, the cryo-electron microscopy structure of the YiiP homologue from *Shewanella oneidensis* is available in its inward-facing (IF) state with the two TM3–TM6 forming an extensive dimeric interface in the transmembrane region.^31^ The comparison of the three-dimensional (3D) structure of the inward- and outward-facing states suggested a zinc-for-proton exchange relying on the so-called alternating mechanism that requires the switch between the two states.^32^ This conformational switch is triggered by an allosteric mechanism that connects the CTD to the TMD through a zinc-dependent reorientation of the TM3–TM6 helix pair.^30^ Notably, CTD-truncated versions such as ZitB from *E. coli* and CzcD from *Cupriavidus metallidurans* can still transport zinc, although with a decreased activity.^33^ Recent studies of the inward-facing state of YiiP suggested that a motion of the four-helix bundle relative to the static TM3–TM6 scaffold is sufficient to allow zinc transport.^34^ This is known as the rocking-bundle mechanism and is present also in other secondary active transporters.^35,36^ MD simulations revealed that these conformational motions occur upon zinc binding to the transport site without breaking the dimer interface.^37^ Overall, these findings suggest that different CDF members might adopt different mechanisms for zinc transport. In this context, biophysical studies on the CTD of the R325 and W325 variants of ZnT8 revealed small discrepancies between the two variants, as well as a different zinc-binding stoichiometry between ZnT8 and YiiP.^38^ This might underlie distinct transport mechanisms and environmental conditions in which the two proteins operate, since the bacterial protein exports an excess of zinc, while there is no evidence for an excess of zinc in the cytosol of eukaryotic cells when exporting into secretory granules.^39,40^ Thus, only ZnT8 has to work against a concentration gradient.

Here, we explored the conformational dynamics of the R325 and W325 variants of the human ZnT8 by MD simulations of the inward-facing state in the presence of zinc(II) ions. We observed that the channel of the two variants had a similar attraction for the metal ions. Instead, the zinc coordination in the transport site was different. The W325 variant appeared to be quite rigid, whereas the R325 variant sampled significant conformational motions. The overall dynamics was accompanied by coordinated CTD motions. Our work highlighted some clear differences in the overall dynamics of the two most common variants of human ZnT8 in the presence of zinc ions.

## Methods

Because the first cryo-EM structures of human ZnT8 have been released^24^ when this paper was already in revision, we exploited the YiiP cryo-EM structure from *S. oneidensis* (PDB ID: 5VRF)^34^ to build a homology model of the dimeric model of the inward-facing (IF) state of human ZnT8. The structural alignment between our IF ZnT8 model and the experimental IF subunit (PDB ID 6XPF chain A, solved at 5.90 Å resolution) is very good (Figure S1), with a root-mean-square deviation (RMSD) between the two aligned TM domains and the two CTD domains of 1.9 and 2.3 Å, respectively. Our model was generated by providing the template and coevolution contacts as distance restraints to the I-Tasser server.^41^ The coevolution contacts were computed with Gremlin, which adopts an unsupervised method to predict residue–residue contacts.^42^ The protein was capped to the N- and C-termini of each chain with the acetate (ACE) and *N*-methyl (NME) caps, respectively. The first model built was the protein in its most active and most frequent variant R325. To build the less active variant, we duplicated the R325 protein and mutated the arginine residue in position 325 to a tryptophan (W325).

The membrane builder module of the CHARMM-GUI was used to embed the protein in a rectangular lipid bilayer composed of a mixture of 1,2-dioleoyl-*sn*-glycero-3-phosphocholine (DOPC), 1,2-dioleoyl-*sn*-glycero-3-phosphoethanolamine (DOPE), and 1,2-dioleoyl-*sn*-glycero-3-phospho-(1′-*rac*-glycerol) (DOPG) with a 2:1:1 stoichiometry.^23,43,44^ The system was then solvated with TIP3P water molecules; 11 zinc ions were added manually in the proximity of the CTD by substituting water molecules, and the charge of the system was neutralized with NaCl. All of the histidine residues were kept neutral and protonated on the Nε tautomer. All of the simulations exploited the same force fields (FFs) for the lipid and the protein portions of the system, the Amber LIPID17 and the Amber ff14SB, respectively.^45,46^ The calculations were performed with the AMBER molecular dynamics (MD) package using the pmemd software,^47,48^ with the same equilibration protocol. An initial minimization step was carried out using the Steepest Descent algorithm followed by Conjugate Gradient. Langevin dynamics with a collision frequency of 1 ps^–1^ was used to linearly heat the system in constant volume for 1 ns, during which the protein and the ions are restrained with a force constant of 10 kcal/(mol Å^2^). The equilibrium temperature was set to 310 K. The physiological density of the system was achieved by carrying out an NPT simulation in which the anisotropic pressure scaling is controlled by the Berendsen barostat (pressure relaxation time of 2 ps). Covalently bonded hydrogen atoms were constrained with the SHAKE and SETTLE (for water molecules) algorithms. A long-range cutoff of 10 Å was applied to compute electrostatic interactions with the particle mesh Ewald (PME) method. After the equilibration and prior to the production run, a brief MD of 2 ns was carried out to relax the protein and reach convergence. The production runs were performed on Nvidia Pascal Xp GPGPU in the same NVT conditions reported above for the heating step but without restraints.^49^

For each variant, we performed 120 short simulations of 10 ns. Sixty simulations were computed with the compromise set (CMset) of parameters for divalent ions (available from the frcmod.ions234lm_126_tip3p file of the AMBER18 package); these parameters are based on the use of the TIP3P water model in combination with the classical 12-6 Lennard-Jones (LJ) nonbonded model.^50^ The other 60 runs were performed with nonbonded parameters for the zinc(II) ion and its protein ligands incorporating polarization effects (newFF set).^51^ In the R325 runs, the latter partial charges and LJ parameters were applied to the residues previously predicted and recently observed to coordinate the zinc ion in the transport site of human ZnT8 (D110–D224–H106–H220).^24,52^ In the W325 runs, the parameters of residues E88 and D103 were additionally changed to the newFF set.

The last frame of three short CMset runs was used as the starting point for three different long simulations. For the W325 and R325 variants, we extended run 5 and run 31, respectively, both featuring a zinc ion in the channel. Moreover, for the R325 variant, we elongated an additional run (run 17) that did not present any zinc ion in the channel, hereafter called R325-NoZinc. The R325-NoZinc was carried out for 800 ns. Instead, the two holo simulations were carried out for 2 μs, of which the first half as classical MD and the second half as accelerated MD (aMD). The bias potentials to be applied, both the whole protein and the dihedrals, were calculated by averaging the potential energy in the first half of the trajectory. The resulting values and the inverse strength boost factors for the total and dihedral potential energy can be found in the “prod.in” file deposited in the Zenodo database (see below). Among the newFF W325 short runs, we extended run 14 and run 15 until 200 ns and run 56 until 1 μs. Instead, among R325 runs, run 27 was extended to 1 μs. In this simulation, the zinc distance from the coordinating atoms was restrained with a flat-based potential having a null force in the range of 2–2.2 Å for 2 ns, to drive the zinc(II)–donor distances within a reasonable range, and then removed. After the removal, the Zn–Asp and Zn–His distances converged to 1.8 and 1.9 Å, respectively.

RMSD values were calculated on the protein Cα atoms from the starting conformation. Distance calculations as well as the radial distribution function (RDF) were computed on the zinc coordinating atoms. The distance between V219 and I266 was calculated between the centers of mass of the atoms of the side chain. The root-mean-square fluctuation (RMSF) in the long CMset runs was computed only in the second half of the trajectories, where convergence was clearly achieved. The RMSF of the TM helices and the CTD were computed separately by fitting the target residues on the average trajectory coordinates in turn calculated by fitting all frames on the Cα, C, and N atoms of the first frame.

Although the core of this work is based on the model built on the bacterial template, we also generated a full-length model using the human ZnT8 cryo-EM structure (PDB 6XPF) as a template. The template, and consequently the final model, has a hybrid channel configuration composed of one channel in the IF state and the other one in the OF state. The first 49 residues missing in the template structure were built ab initio with Quark.^53^ The whole model, including the four zinc ions coordinated at the CTD, was built with RosettaCM.^54^ The protein side chains were repacked and minimized with the fastRelax mover in Rosetta.^55^ The missing loops were modeled by selecting the lowest energy conformation generated with the kinematic loop modeling protocol in Rosetta.^56^ The protein was then embedded in the membrane and equilibrated as described above. One classical simulation of 500 ns was performed for each variant with the parameters reported above; in particular, the parameters used in the newFF simulations were applied to all of the residues coordinating a zinc ion in the experimental structures.^24^

All models, MD trajectories, and parameter files are freely available from the Zenodo website at url (DOI: 10.5281/zenodo.4381998).

## Results

The dimeric model of the ZnT8 protein in the inward-facing (IF) state was built using the YiiP inward-facing state (PDB ID: 5VRF) as a structural template. To improve the prediction of the structure of the cytosolic domain of ZnT8, we included coevolution contacts computed with an unsupervised method as distance restraints in the I-Tasser fragment assembly procedure. The resulting model had an RMSD from the experimental structure of the subunit in the IF configuration (PDB ID 6XPF, chain A) of 2.0 Å over the entire subunit (Figure S1).

**Figure 1:**
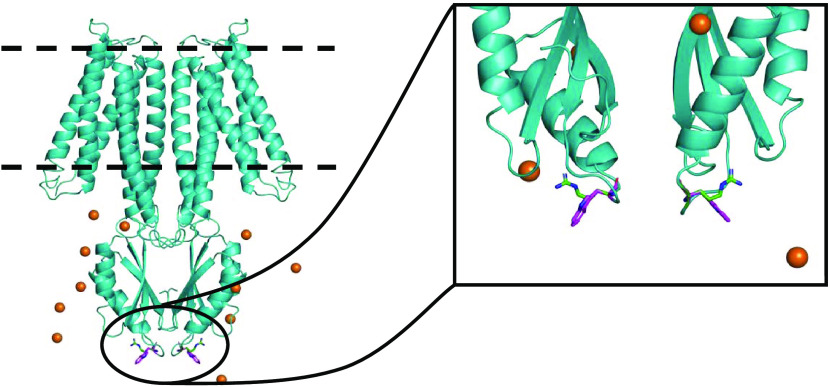
ZnT8 model embedded in the membrane (dashed lines). Zinc(II) ions are shown as orange spheres. The tryptophan and arginine residues 325 are shown as magenta and green sticks, respectively.

The dimeric protein model was embedded in a lipid bilayer, solvated, and 11 zinc(II) ions were added in the proximity of the CTD (Figure 1). By construction, the structural models of the two protein variants were identical, except for residue 325 (inset of Figure 1). For each variant, we have carried out 60 runs of 10 ns each by applying two different force fields, hence computing a total of 120 short simulations per variant; the FFs used were the CMset for divalent cations and a recent FF (newFF) in which new partial charges and Lennard-Jones parameters were derived to take into account the polarization effects of the cation on the coordinating residues.^51,57^

### Short Simulations with the CM Parameter Set

**Figure 2:**
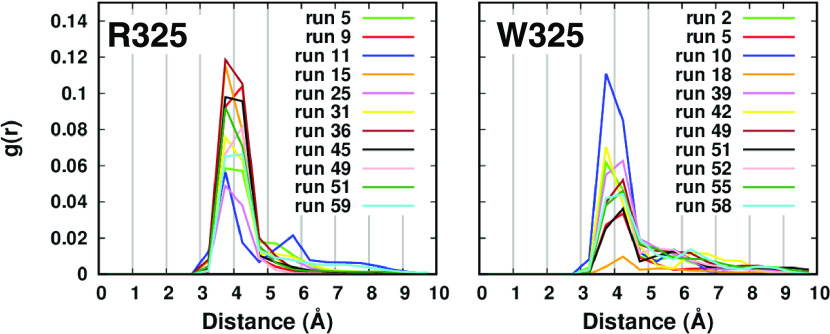
Radial function of the distance between the zinc ion in the channel and the aspartate pair D110–D224 of the transport site.

About 20–25% (13/17 out of 60) of the short simulations featured a zinc ion in one of the two transmembrane channels at the end of the trajectory. Instead, none of them presented a zinc ion in both channels simultaneously. The percentage of runs with a zinc ion in the channel was similar between the two variants, suggesting a comparable affinity for the metal. In most cases, the zinc ion was bound within the transport site. The radial function of the distance between the zinc ion in the channel and the aspartate residue pair D110–D224 of the transport site reveals that 11 runs for each variant sampled a detectable zinc interaction in the transport site (Figure 2). The R325 variant has higher peaks than W325, suggesting that this interaction might be less stable in the less active variant.

**Figure 3:**
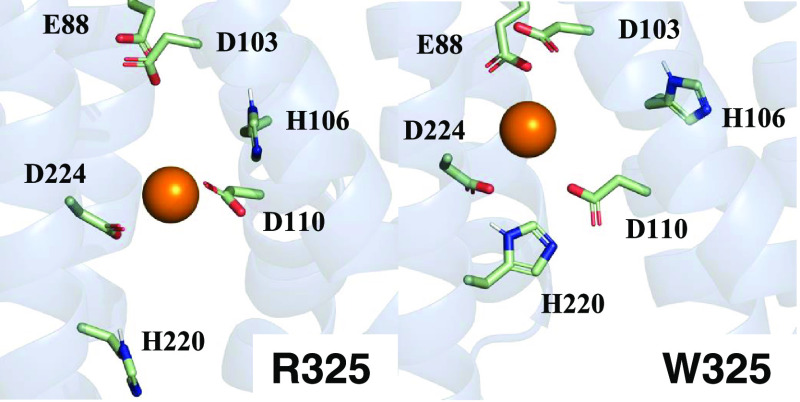
Zinc coordination in the transport site.

The comparison of the two snapshots of the transport site region at 10 ns, one for each variant (Figure 3), pointed out a slightly different metal environment between the two variants. Indeed, the zinc ion in the R325 snapshot is in the proximity of only the residue pair D110–D224. Instead, in the W325 frame, the zinc ion is close also to E88–D103, in addition to D110–D224, thus identifying a pocket above the transport site, i.e., closer to the top exit of the channel.

**Figure 4:**
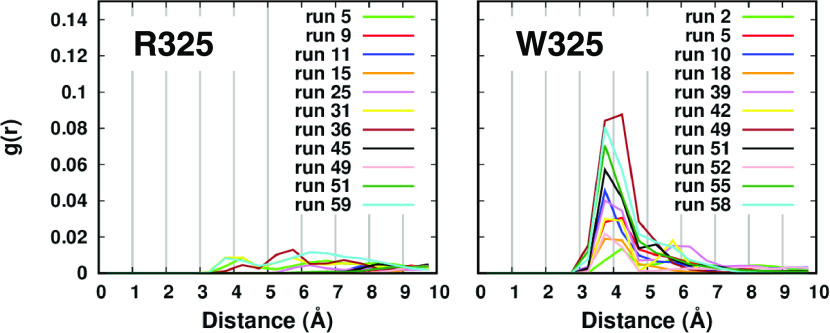
Radial function between the zinc ion in the channel and the residue pair E88–D103.

We assessed whether the E88–D103–D110–D224 pocket was consistently populated by computing the radial function between the zinc ion and the residue pair E88–D103 in all of the 11 simulations where the zinc ion reached the transport site (Figure 4). The presence of peaks only in the W325 panel shows that the pocket is accessible only in this variant, albeit to a different extent among the runs.

**Figure 5:**
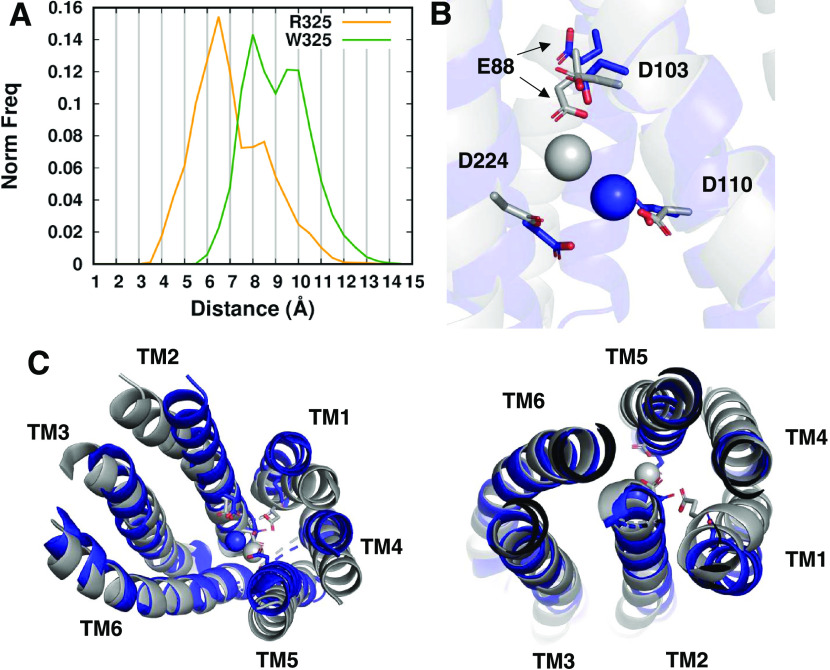
Structural differences in the channel of the two variants. (A) Normalized frequency distribution between D110 and D224 performed on the runs coordinating a zinc ion in the transport site. (B) Zinc coordination in the two variants. The fitting was performed on the Cα atoms of TM3 and TM6. (C) Structural alignment of the TM helices in the channel. Bottom view on the left, top view on the right. Gray = W325, blue = R325.

To investigate the reasons for the different zinc environments between the two variants, we computed the normalized frequency distribution of the distance between the transport site residues D110 and D224, which are located, respectively, on the TM2 and TM5 helices, for the same 11 runs (Figure 5A). On average, the W325 runs sampled higher values than those of R325. Figure 5B shows the superimposition of the channels of the two variants, computed by fitting the long TM3–TM6 helix pair. The position of D110 is maintained in the two variants, whereas D224 is displaced and is more distant from D110 in W325. Moreover, in W325, the E88 residue on TM1 is positioned closer to the D110–D224 pair (Figure 5B).

The bottom and top view of the TM helices show a different arrangement of the four-helix bundle TM1–TM2–TM4–TM5 between the two variants (Figure 5C). In particular, TM5 is more distant from TM2 in the W325 variant; the lower part of TM2 itself has a different orientation in the two variants. The latter structural difference explains why D224 is more distant from D110 in W325 than in R325. The higher TM2–TM5 distance in W325 can in turn depend upon the different orientation in the lower/middle portion of TM4–TM5 that causes a different tilt of the helices in the two variants. Consequently, TM1 in W325 is closer to the inner part of the channel than in R325, bringing E88 nearer to the transport site.

In summary, in the W325 variant, the different orientation of TM4–TM5 results in a larger distance of D224 from D110 and makes the upper part of the channel less packed than in R325. These two phenomena, together with the repositioning of TM1, may be responsible for the formation of the E88–D103–D110–D224 pocket and the access of zinc to it.

### Short Simulations with the newFF Parameters

**Figure 6:**
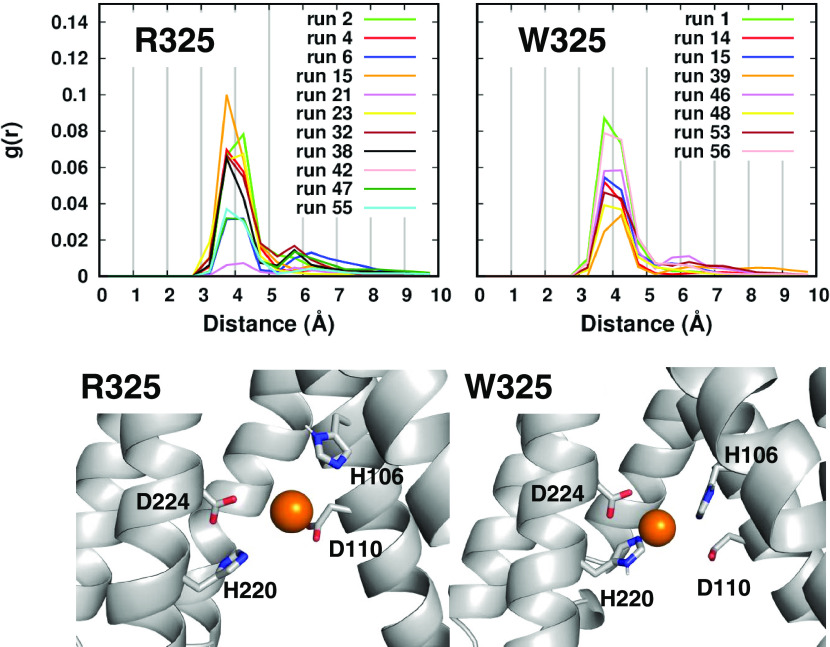
Radial function of the distance between the zinc ion in the channel and the four residues of the transport site. The bottom panels show the zinc environment in the transport sites of run 27 and run 56 for the R325 and W325 simulations, respectively.

Sixty runs of 10 ns were performed for each variant using a recent FF, developed to take into account the polarization effects of the zinc(II) on the coordinating residues.^51^ The radial function of the distance between the zinc ion in the channel and the transport site residues reveals that 11 and 8 runs sampled an interaction in the R325 and W325 variants, respectively (Figure 6). At variance with what was observed with the CMset, here both variants sampled a metal environment involving all of the four residues of the transport site, including the two histidine residues H106–H220 (Figure 6).

**Figure 7:**
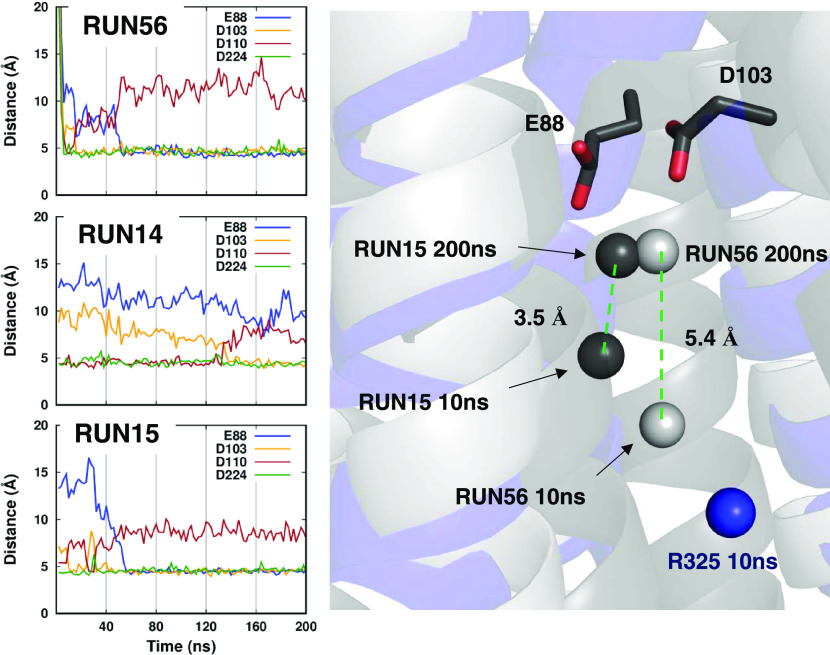
Translocation of the zinc ion in the W325 newFF simulations. The distance between the zinc ion in the channel and the surrounding negatively charged residues is shown on the left. D110 and D224 belong to the transport site, E88 and D103 belong to the pocket above the transport site. To better appreciate the difference in behavior with respect to the R325 simulations, the position of the zinc ion from one of these runs at 10 ns is shown (blue sphere).

To verify whether the W325 variant could sample the same pocket observed with the CMset, we extended three runs and measured the zinc distance from the group of residues D110–D224 of the transport site and E88–D103 located above the transport site (Figure 7). The zinc ion behaved similarly in all three runs, translocating from the transport site to the pocket above it. In this process, the metal lost contact with D110, whereas D224 remained at a stable distance. By superimposing the TM helices of the three runs, one can compare the position of the zinc ion in the channel. Notably, at 10 ns, the zinc position in the W325 runs is already significantly deeper in the channel than for R325 (Figure 7). In the time range from 10 to 200 ns, the metal ion covered 3.5 and 5.3 Å in run 15 and run 56, respectively, eventually reaching E88 and D103.

In summary, with the newFF force field, some simulations sampled zinc coordination involving all of the four residues belonging to the transport site as observed in the cryo-EM structures of ZnT8. Some of the W325 simulations featured zinc coordination in a pocket above the transport site, defined by residues E88–D103–D110–D224, similarly to what was also observed with the CMset but on a longer time scale.

### Long Simulations

We carried out four long simulations for each variant, two of 2 μs with the CMset and two of 1 μs with the newFF. In the CMset simulations, the first half of each trajectory was performed by applying a classical MD method, instead, the second half was accelerated by applying a biased potential that enhanced the conformational sampling. In addition, we performed 800 ns of classical MD starting from an R325 frame without zinc ions in the channel (named R325-NoZinc). The RMSD of the long trajectories shows that convergence was reached in all of the simulations (Figure S3).

**Figure 8:**
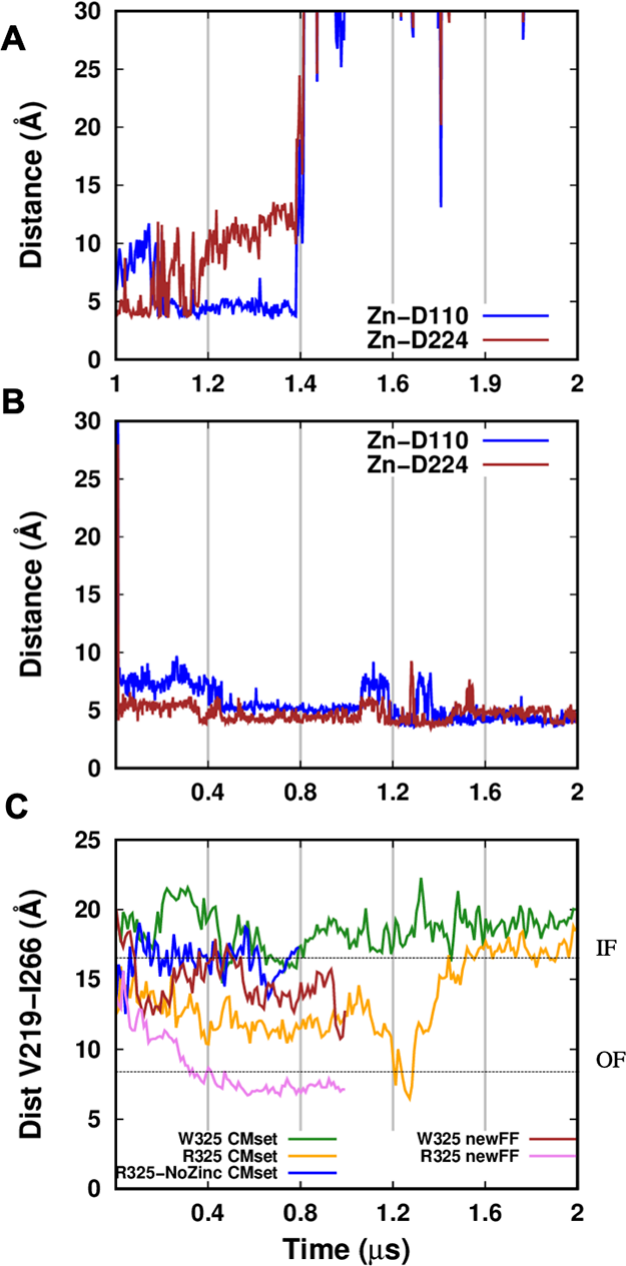
Conformational changes upon zinc interaction in the transport site. (A) Distances between the zinc ion in the channel and the two transport site aspartate residues D110 and D224. (B) Distance between V219 (TM5) and I266 (TM6) in the second half of the R325 and W325 simulations. (C) Distance between V219 (TM5) and I266 (TM6) during the long simulations and in the cryo-EM structures^53^ (dashed lines, IF = inward-facing, OF = outward-facing).

As observed in the short simulations, the radial function of the zinc distance from either D110–D224 or E88–D103 reveals that in the R325 variant with the CMset, the zinc ion interacted only with the residue pair in the transport site (D110–D224) (Figure S4). Instead, in the W325 variant the zinc ion interacted with both pairs. Various movies were collected to show the main structural features sampled by these simulations. First, the movie of the zinc permeation in the channel of the W325 variant shows the metal ion translocating directly from the cytosol to the pocket above the transport site, interacting with all four residues (E88–D103–D110–D224) simultaneously (Movie S1). Instead, the corresponding movie for R325 shows the zinc ion binding only the transport site residues D110–D224 (Movie S2). Interestingly, in the second half of the R325 simulation with the CMset, the zinc ion leaves the transport site to move back to the cytosol. To verify this, we measured the distance between the zinc ion and the transport site residues D110–D224 along time (Figure 8A). This analysis shows that the metal ion first loses contact with D224 and afterward with D110 at about 1.4 μs. The same analysis of the W325 simulation shows that the zinc ion remained in the channel (Figure 8B). The relationship between the zinc release in the R325 simulation and the channel dynamics was then correlated to the distance between V219 and I266 (Figure 8C). These two residues are located in the middle of TM5 and TM6, which are the two helices constituting the front line of the channel gate. The experimental distances between V219 and I266 taken from the recent cryo-EM structures of ZnT8 in the IF and OF states^24^ are shown in the plot as dashed lines. In the R325 simulation with the CMset, the channel started to close shortly after the first wide zinc fluctuations (between 1 and 1.2 ns), ending up with a complete channel closure. Once the channel is closed, the V219–I266 distance in the simulation and in the OF state channel is very similar. Subsequently, the zinc ion is released and the channel opens again, reaching a width comparable to that of W325 as well as of the R325-NoZinc simulation, and also quite similar to the cryo-EM structures in the IF state (Figure 8C). The distances observed in the trajectories sampled with the newFF are shorter than those observed with the CMset. In particular, for the R325 variant, where we pushed the zinc ion to closely approach the interacting residues, the trajectory converged to a V219–I266 distance very similar to that measured in the experimental structure in the OF state.

The movie of the zinc release in the R325 CMset simulation shows that V219 and I266 get in contact at the moment of the maximum closure of the channel (Movie S3). During the overall process of closing and reopening, the helices of the four-helix bundle rotate and tilt, ending up with a different orientation that can be described as a switch from a tilt to the left to a tilt to the right with respect to the TM3–TM6 helix pair.

**Figure 9:**
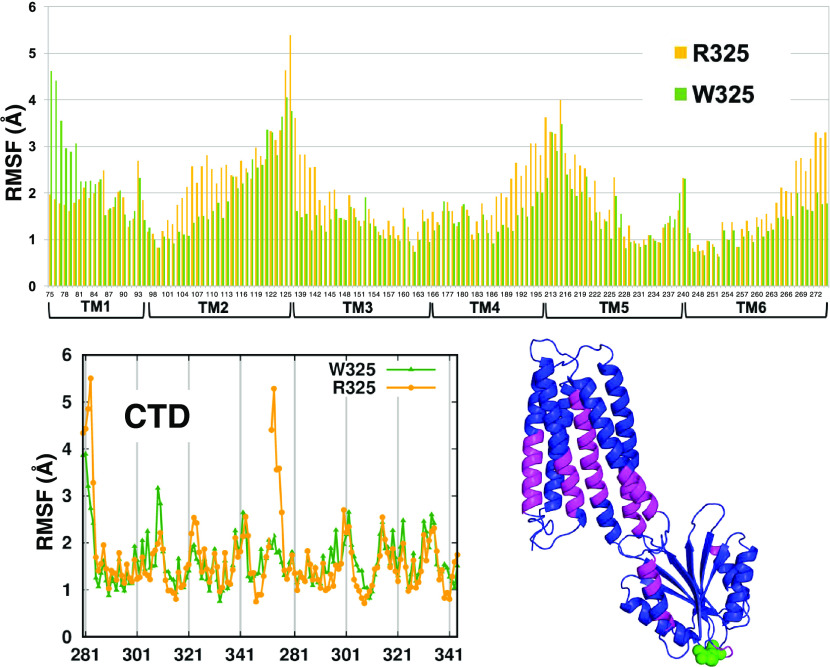
RMSF of the TM helices in the channel affected by zinc binding and in the CTD. The protein regions where R325 has the highest difference from W325 are colored in magenta in the 3D structure.

The dynamics of the whole protein dimer in the long CMset simulations can be visualized in the movies of the full trajectory (Movies S4 and S5). In general, the W325 variant appears more rigid than the R325 variant where, on the contrary, the protein undergoes remarkable conformational motions. This is also documented by the RMSF of the TM helices (only in the channel with the zinc ion) and the CTD (Figure 9). In particular, the W325 variant has a constantly open channel in the presence of the zinc ion (Figure 8C) and a CTD stuck in the starting orientation (Movie S4). Instead, as already described, in the R325 variant the channel closes upon zinc binding in the transport site and becomes accessible again when the metal ion returns to the cytosol (Movie S5). Interestingly, the movie points out that this rearrangement is accompanied by two main conformational motions of the CTD, which somehow have complementary behavior during the “channel closing” and “channel reopening” steps. In fact, the CTD moves toward the channel experiencing the closing process while this is ongoing and, instead, moves toward the other empty channel during the reopening. Accordingly, in the newFF R325 simulation, the CTD moves toward the channel that becomes closed (Movie S6), whereas in the newFF W325 simulation the CTD is fluctuating around the starting orientation (Movie S7).

The comparison of the RMSF values shows that all of the TM helices of R325 are more flexible than those of W325, except for the first portion of TM1 that constitutes the N-term of the protein. The highest difference is in the first portion of TM2, TM3, and TM5 and in the last portion of TM4 and TM6. Within the CTD, the largest difference is in the small helix that connects the CTD to the TM6 helix and in three residues (322–323–324) next to the variation site. The mapping of the residues that are more flexible in the R325 variant on the protein structure (colored in magenta in Figure 9) shows that these regions are close in space. Therefore, the higher flexibility induced by the R325 mutation may transfer from the CTD to the TM helices through this pathway, suggesting a more efficient communication from the CTD to the transport site in R325 than in W325. Notably, the channel not affected by zinc permeation features a very low flexibility in both variants (Figure S4).

**Figure 10:**
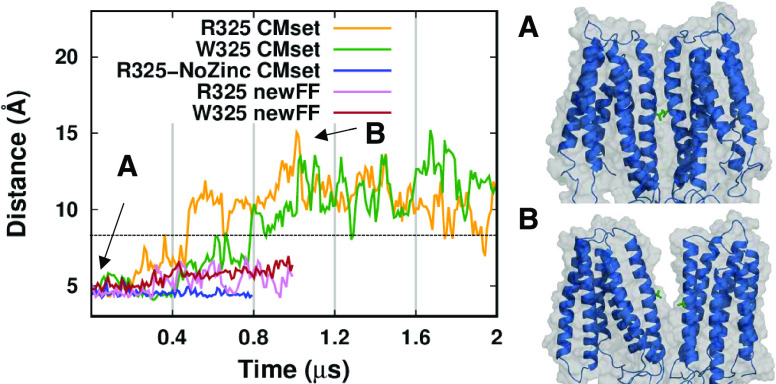
Dimer interface dissociation along the simulations. The distance was measured between the V153 (TM3) residue of the two subunits, shown as green sticks in the protein structures on the right. The dashed line represents the corresponding distance in the cryo-EM structures.^53^ (A) Snapshot at the beginning of the simulation. (B) Snapshot at 1 μs.

From the movies, it is possible to observe a partial separation of the two transmembrane channels along their dimeric interface. To characterize this process, we monitored the distance between the residue V153 located on each monomer in the middle of the dimer interface (Figure 10). At the starting point of the simulations, the two V153 residues are in close contact. Then, they start to split apart at 200 ns in the R325 simulation and 200 ns later also in the W325 run. After a constant and similar increase in the first half of the simulations, in the second half of the W325 run, the distance seems to continue to increase, even if more slowly and with wide oscillations. On the contrary, in the R325 run, the distance reaches a plateau of 12 Å from 1 to 1.4 μs and then, at this simulation time, i.e., when the zinc ion leaves the channel, the two residues slightly reduce their distance. Of note, in the R325-NoZinc run, the dimer interface remains intact throughout the whole 800 ns of simulation. In the newFF simulations, the distance increases less than in the CMset simulations. For reference, in the cryo-EM structures the dimeric interface does not change with the structural state of the channel, the V153 distance being 8.3 Å in all of the deposited models.

In summary, the zinc environment observed in the short simulation is conserved in the long simulations. The W325 protein binds the zinc ion in a site composed of the residue pair E88–D103 and the D110–D224 residues from the transport site. The trajectories point out that the W325 variant is more rigid than the R325 variant, which instead shows relevant conformational motions, such as the closing of the channel after zinc binding to the transport site. The RMSF analysis indicates that the largest difference in dynamics occurs within the channel affected by zinc permeation and in a small helix of the CTD that, intriguingly, is located in direct contact to the mutation and connects the CTD to TM6 through a loop. Finally, we observed that after the zinc ion reached the transport site, the dimer interface started to dissociate in both variants. However, this dissociation process was less evident in the newFF simulations and did not take place in the R325-NoZinc simulation where the zinc ions did not enter the protein channel.

### Simulations of the Full-Length Model (Based on the6XPFStructure)

For each variant, we carried out one simulation of 500 ns with the zinc ions bound to the CTD, as proposed in the 6XPF structure,^24^ and 11 free zinc ions in solutions. In these simulations, none of the free zinc ions permeated the accessible IF channel. The conformational dynamics of the structures along time was assessed by calculating the RMSD from the starting conformation separately for the TMD and the CTD (Figure S6). In this regard, the W325 TMD appears more stable than R325, with the latter featuring greater fluctuations (Figure S6A). The CTD is more stable than the TMD in both the variants, with the conformation of R325 being more distant from the starting model than that of W325 (Figure S6B). The superimposition of the TMD conformation at 500 ns and the starting structure shows that the IF channel is open in the R325 variant but closed in W325 (Figure 7A). Interestingly, in the R325 variant, the OF channel sampled large helical motions that moved it away from the IF channel (Figure 7B). The same superimposition shows that the CTD is more tilted toward the OF channel in the R325 variant with respect to W325 (Figure S7A,B respectively). To visualize the main protein motions, we recorded a movie of the R325 trajectory showing the significant flexibility of the open IF channel while the CTD is moving toward the OF channel (Movie S8). The corresponding W325 movie instead shows the channel closure, while the CTD slightly rotates on its *Y* axis (Movie S9).

**Figure 11:**
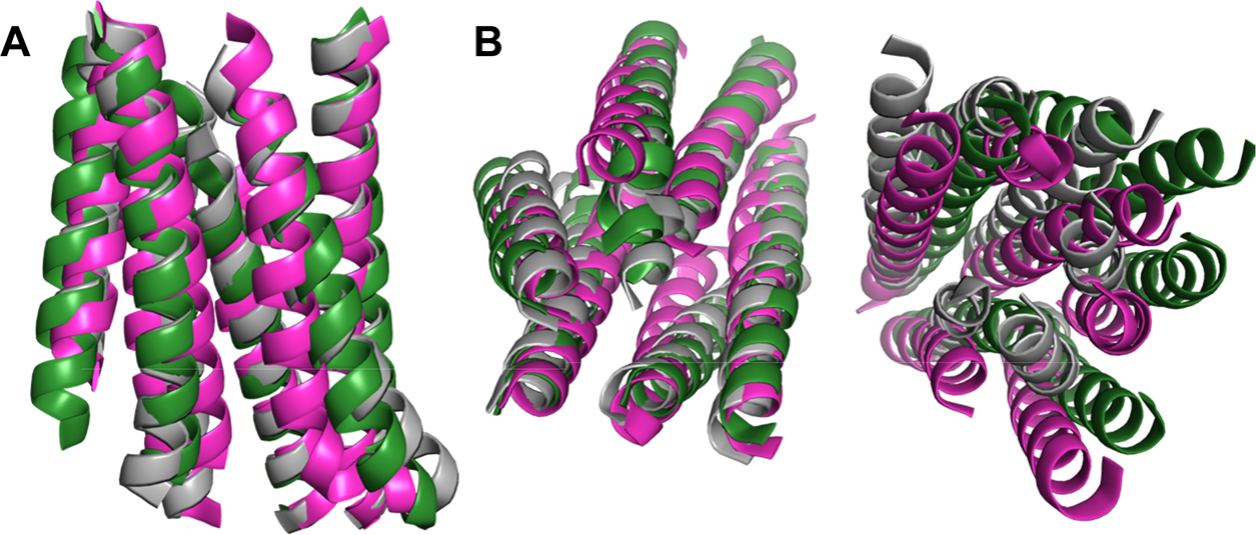
Superimposition between the TM channels at 500 ns and the starting model. The starting model is shown in gray, the R325 and W325 models at 500 ns are shown in green and in magenta, respectively. (A) IF channel. (B) Top view of the whole TMD.

In summary, the full-length model trajectories show dynamical properties similar to the ones observed in the previous simulations where the model was built on the bacterial template. In fact, the W325 in general appears more rigid than the R325 variant in which the IF channel is always open and accessible. The enhanced flexibility of the TMD region of the R325 variant also resulted in a large rearrangement of the OF channel (Figure 11).

## Discussion

In this study, we built a model of the whole ZnT8 dimeric protein in its IF state to investigate by MD simulations how the two most common variants in the human population, R325 and W325, interact with zinc(II) ions. Previous work on the kinetics of zinc transport by ZnT8 suggested that activity depends on the lipid environment, concluding that a mixture of DOPC, DOPE, and DOPG at a 2:1:1 ratio yielded the top *V*_max_.^23^ With such a lipid composition, which is close to that of insulin secretory granules, the R325 variant exhibited accelerated zinc transport kinetics with respect to the W325 variant. Thus, we used the same ratio of these three classes of lipids in our simulations. Recently, cryo-EM structures of human ZnT8 have been released, showing the protein in both the full OF state and in a hybrid configuration with one channel in the IF state and the other in the OF state.^24^ Despite our model was built using only the bacterial YiiP as a template, the IF channel is very similar to the ZnT8 experimental structure (Figure S1).

One remarkable difference between the two variants in our simulations was in the position of zinc within the transport site. The active R325 variant displayed zinc bound in the transport site. With the CMset parameters, the zinc ion interacted only with residues D110 and D224, which are conserved among all ZnT members and in YiiP.^27^ Replacing D110 and D224 with asparagines indeed abrogates zinc binding in this site.^24^ The process by which the zinc ion permeated the open channel from the cytosol and approached the transport site is analogous to what we observed for the bacterial homologue YiiP.^37^ However, zinc coordination in the transport site of ZnT8 also involves residues H106 and H220,^24^ which are conserved in all ZnT members, except in ZnT6, ZnT9, and ZnT10.^27^ By applying new parameters^51^ for zinc–His and zinc–Asp interactions, we successfully reproduced the zinc coordination in the transport site of the R325 variant. Instead, with both parameter sets, we observed that for the less active W325 variant, the zinc ion reached a pocket delimited in the lower part by the two aspartate residues of the transport site and in the upper part by E88 and D103. Despite the zinc ion arrived at the same final position in the CMset and the newFF simulations, its trajectory within the channel was somewhat different from the two force fields. In the CMset runs, the zinc ion reached directly into the pocket above the transport site, and in the newFF runs, it first bound in the transport site and then diffused to the pocket above it. Interestingly, similar to the H106 and H220 pair on TM2 and TM5, residues E88 and D103 are conserved in all of the ZnT members, except for ZnT6 and ZnT9.^4^ Moreover, site-directed mutagenesis of the homologue ZnT2 protein identified the same E88 and D103 as the two residues most likely interacting directly with zinc during its transport activity.^58^ ZnT2 transports zinc from the cytoplasm into intracellular vesicles and carries a neutral threonine in position 325, hence having biophysical features more similar to W325 than to R325 in that region. Thus, residues E88 and D103 can be crucial for zinc transport also in the W325 variant of ZnT8, as reported for ZnT2.

The different zinc coordination between the two variants reflects a different rearrangement of the TM helices occurring already in the 10 ns of the short simulations. In this regard, a number of observations in the long simulations pointed to the R325 variant being more flexible and more responsive to zinc binding in the channel than the W325 variant. Indeed, the latter maintained the same channel conformation throughout the trajectory, with the channel open and accessible even after zinc permeation. On the contrary, after the zinc ion reached the transport site, the channel of the R325 variant became narrower, eventually leading to a closure.

Since the two variants differ for a CTD residue and feature different zinc transport activity, one would expect that the mechanism of function of the ZnT8 protein involves allosteric communication between CTD and TMD. However, how this communication works is still unknown. Here, we observed that the CTD of the R325 variant moved toward the channel permeated by the zinc ion and then moved in the opposite direction after the same ion returned to the cytosol and the channel reopened. Instead, in all of the W325 simulations, the CTD was rather stable in its initial configuration. Thus, we observed a correlation between the dynamics of the TMD and the CTD in the R325 protein. A communication between TMD and CTD was also suggested by the RMSF analysis, which showed a highly flexible R325 protein in most of the TM helices and in part of the CTD, whereas the W325 protein was more rigid. The highly flexible regions in the R325 could be part of an allosteric communication pathway that connects the region around residue 325 with the transport site.

During the revision of this article, a structural investigation of human ZnT8 by cryo-EM methods was published, providing three structures solved under different conditions.^24^ The cryo-EM structures revealed two sites occupied by zinc ions, site B formed by H137-H345, and site C, which is located on the CTD. Site B is occupied by zinc ions only in the fully OF structure, whereas in site C two zinc ions are bound to a sequence stretch involving residues from an HCH motif in the N-terminal region as well as C361 and C364 from the other subunit. The latter long C-terminal loop is truncated at D360 in our YiiP-based model. These observations might explain why we never found zinc ions bound to site B or the CTD in our simulations. We modeled the full-length protein using 6XPF as the template, including the initial 49 amino acids that are missing in the experimental structure, and performed two simulations of 500 ns with zinc stably bound to the CTD. Notably, the6XPFstructure, and therefore our model, features one of the channels in the IF state and the other in the OF state. For this system, our simulations did not sample any zinc permeation in the IF channel. These fundamental differences prevent a detailed comparison with the previous simulations. Nevertheless, the simulations with the new model confirmed that the R325 variant is significantly more flexible than the W325 variant. Similar to what is observed in the R325-NoZinc CMset simulation, the R325 IF channel is always open and accessible, whereas the W325 IF channel sampled a closure. An additional structural and experimental investigation of human ZnT8 appeared in 2020,^59^ which suggested that the removal of the N-terminal part of the protein does not affect protein dimerization or the transport activity significantly. Instead, the deletion of the HCH motif markedly reduces zinc uptake activity.^24^ Site C is presumably the site with the highest affinity for zinc in ZnT8, as it is the only one populated with the metal in preparations without added zinc.^24^ It is therefore unlikely that zinc ions can migrate from site C to the transport site, suggesting a regulatory role for the HCH motif rather than its direct involvement in the transport mechanism. In this frame, it is quite relevant that all our simulations, including those starting from the model of the protein based on the cryo-EM structure of human ZnT8, point to the R325 variant being more flexible than W325. The regulatory role of site C could also explain why in the latter runs no zinc ions entered the channel within 500 ns of simulation.

Previous simulations^37^ and experimental data on the bacterial YiiP transporter indicated that this protein can transport zinc while the membrane maintains a persistent dimer interface through the so-called rocking-bundle mechanism, in which the four-helix bundle formed by TM1–TM2–TM4–TM5 rearranges with respect to a static TM3–TM6 helix pair.^34,37^ In line with this, the IF and OF channels feature the same conformation for the TM3–TM6 pair in the cryo-EM structure.^24^ Our simulations on ZnT8 showed instead that in both the variants the dimer interface became looser upon zinc binding to the transport site, whereas there was no dissociation along the dimer interface in the absence of zinc ions in the channel. This process took place despite our initial model had a dimeric interface significantly more compact than the experimental structures and can possibly reflect a difference in the details of the transport mechanism between the human and bacterial transporters.

## Supporting Information Available

Superimposition of the TM regions of our ZnT8 model based on the bacterial YiiP structure and of the cryo-EM structure of human ZnT8 in the IF state (Figure S1); zinc coordination in the R325 transport site before and after the application of distance restraints (Figure S2); RMSD of the long simulation trajectories (Figure S3); radial function of the distance between the zinc ion in the channel and its coordinating residue pair in the CMset simulations (Figure S4); RMSF of the TM helices in the channel not affected by zinc binding in the CMset simulations (Figure S5); RMSD of the full-length models built on the 6XPF cryo-EM structure of human ZnT8 along time (Figure S6); superimposition between the CTD at 500 ns and the starting model (Figure S7) (PDF)
Movie of the zinc permeation in the CMset W325 channel (Movie S1) (MPG)
Movie of the zinc permeation in the CMset R325 channel (Movie S2) (MPG)
Movie of the TM helices dynamics in the CMset R325 channel (Movie S3) (MPG)
Movie of the whole CMset W325 trajectory (Movie S4) (MPG)
Movie of the whole CMset R325 trajectory (Movie S5) (MPG)
Movie of the whole newFF W325 trajectory (Movie S6) (MPG)
Movie of the whole newFF W325 trajectory (Movie S7) (MPG)
Movie of the R325 “full-length 6xpf-based model” trajectory (Movie S8) (AVI)
Movie of the W325 “full-length 6xpf-based model” trajectory (Movie S9) (AVI)

## Supplementary Material

ci0c01139_si_001.pdf

ci0c01139_si_002.mpg

ci0c01139_si_003.mpg

ci0c01139_si_004.mpg

ci0c01139_si_005.mpg

ci0c01139_si_006.mpg

ci0c01139_si_008.mp4

ci0c01139_si_009.avi

ci0c01139_si_010.avi

## Notes

Authors will release the atomic coordinates and experimental data upon article publication.

## Abbreviations

T2D: type-2-diabetes
MD: molecular dynamics
CDF: cation diffusion facilitators
TMD: transmembrane domain
CTD: C-terminal domain
TM: transmembrane
ACE: acetate
NME: *N*-methyl
LJ: Lennard-Jones
PME: particle mesh Ewald
aMD: accelerated MD
