# Supplementary material for: Insights into the Dynamics of the Human Zinc Transporter ZnT8 by MD Simulations
Source: J Chem Inf Model. 2021 Jan 29;61(2):901–12. doi: 10.1021/acs.jcim.0c01139 (PMC8023586; doi:10.1021/acs.jcim.0c01139)
Supplement: Supplementary file 1 — ci0c01139_si_001.pdf [file ci0c01139_si_001.pdf]

Supporting Information

Insights on the dynamics of the human zinc transporter ZnT8 by MD simulations

Davide Sala[†], *Andrea Giachetti*[‡] *and Antonio Rosato*[†,§,]*

[†]Magnetic Resonance Center (CERM), University of Florence, Via Luigi Sacconi 6, 50019 Sesto Fiorentino, Italy.

[‡]Consorzio Interuniversitario di Risonanze Magnetiche di Metallo Proteine, Via Luigi Sacconi 6, 50019 Sesto Fiorentino, Italy.

[§]Department of Chemistry, University of Florence, Via della Lastruccia 3, 50019 Sesto Fiorentino, Italy.

SUPPLEMENTARY FIGURES AND MOVIES

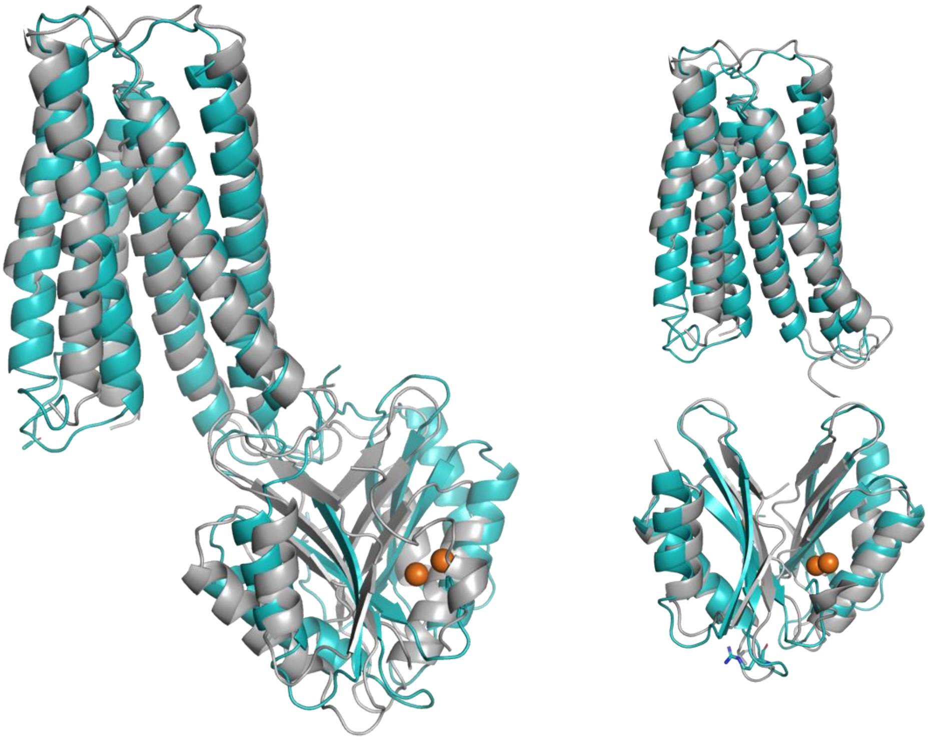

Figure S1. Superimposition on the TM region between our template-based ZnT8 model (cyan) and the cryo-EM structure (PDB ID 6XPF, grey) in the IF state. On the right the TMD and CTD are superimposed separately.

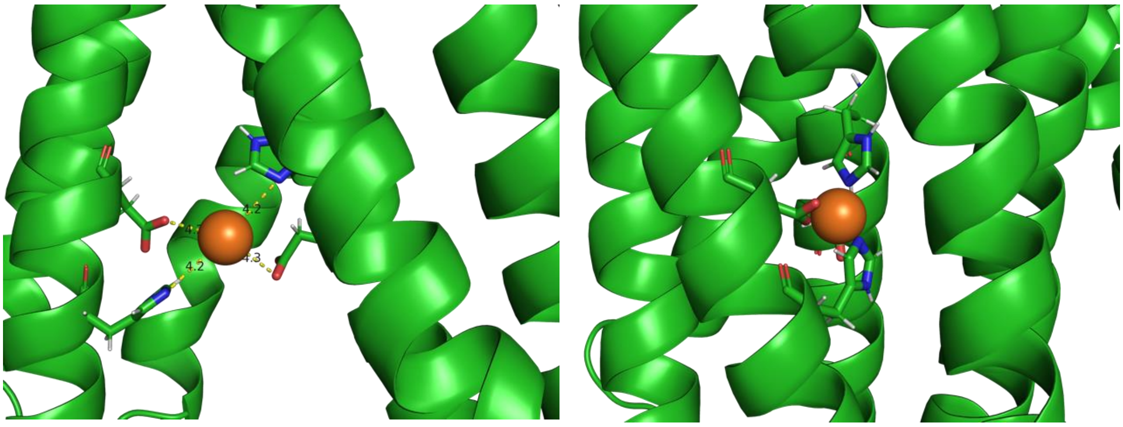

Figure S2. Zinc coordination in the R325 transport site before and after the application of distance restraints.

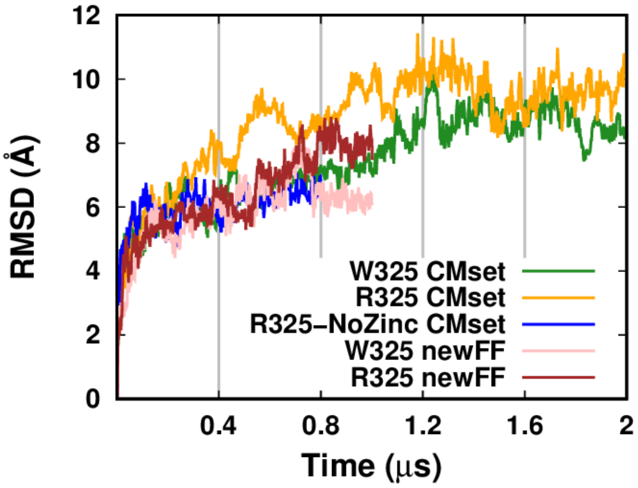

Figure S3. RMSD of the long simulation trajectories.

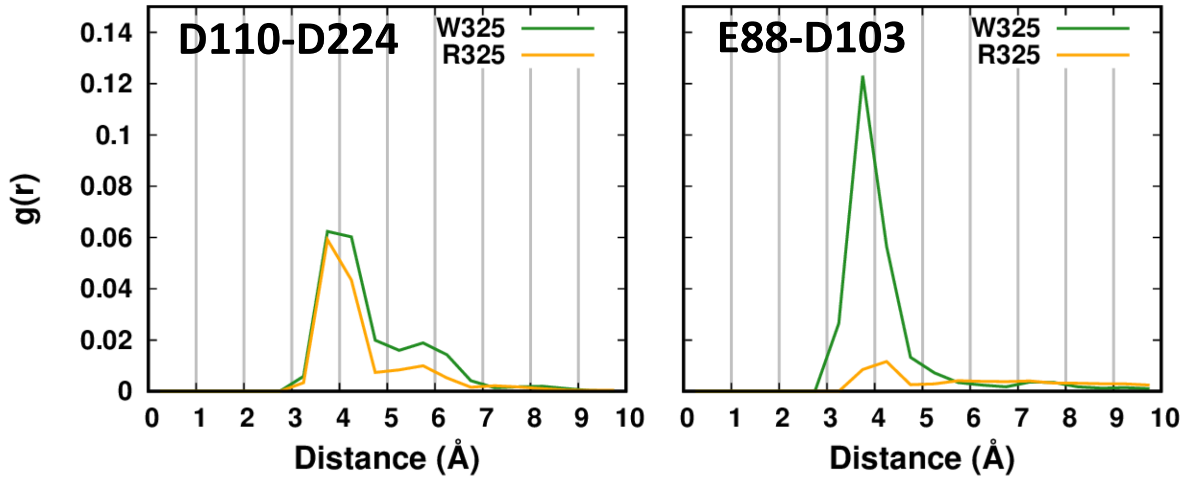

Figure S4. Radial function of the distance between the zinc ion in the channel and its coordinating residues pair in the CMset simulations.

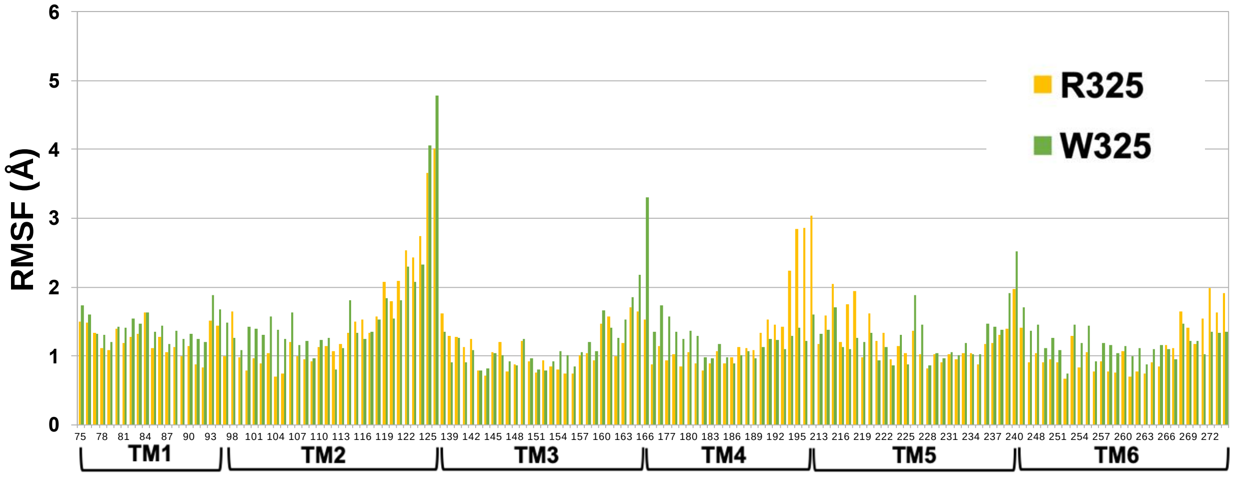

Figure S5. RMSF of the TM helices in the channel not affected by zinc binding in the CMset simulations.

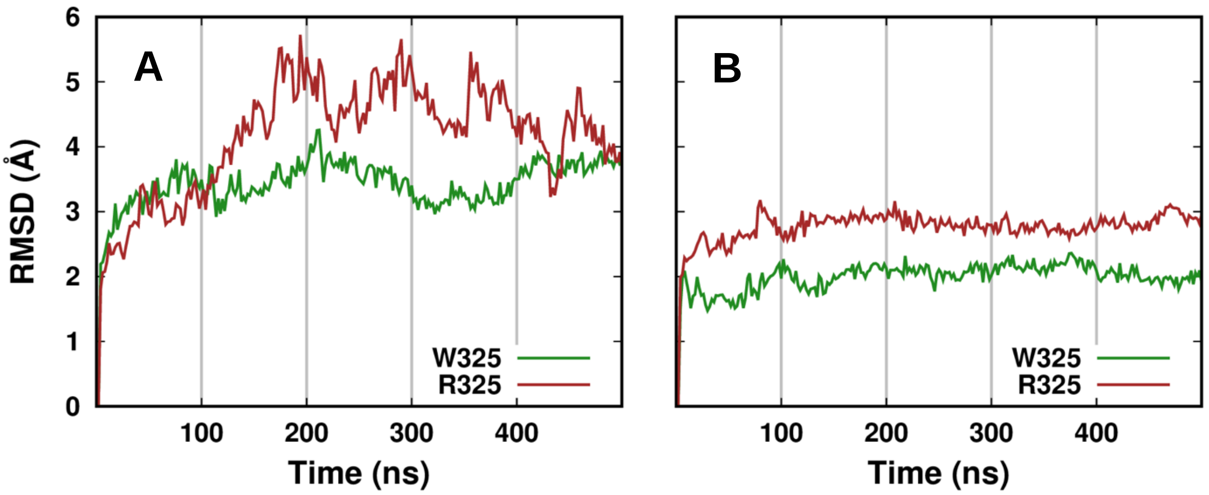

Figure S6. RMSD of the full-length models along time. A) TMD. B) CTD

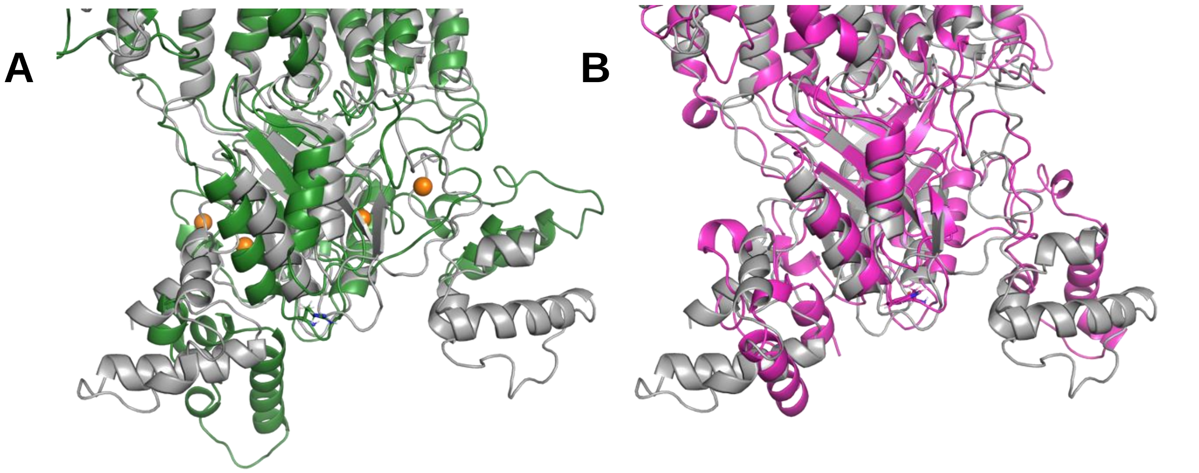

Figure S7. Superimposition between the CTD at 500ns and the starting model. The starting model is shown in grey. A) R325 at 500ns (green). B) W325 at 500 ns (magenta).

Movie S1. Movie of the zinc permeation in the CMset W325 channel (MPG).

Movie S2. Movie of the zinc permeation in the CMset R325 channel (MPG).

Movie S3. Movie of the TM helices dynamics in the CMset R325 channel (MPG).

Movie S4. Movie of the whole CMset W325 trajectory (MPG).

Movie S5. Movie of the whole CMset R325 trajectory (MPG).

Movie S6. Movie of the whole newFF W325 trajectory (MPG).

Movie S7. Movie of the whole newFF W325 trajectory (MPG).

Movie S8. Movie of the R325 “full-length 6xpf-based model” trajectory (AVI).

Movie S9. Movie of the W325 “full-length 6xpf-based model” trajectory (AVI).
